# Pandemic to endemic: A successful transition in Malaysia

**DOI:** 10.7189/jogh.13.03063

**Published:** 2023-12-22

**Authors:** Siti M Abd Gani, Marcel Alied, Nguyen Tien Huy

**Affiliations:** 1 School of Tropical Medicine and Global Health, Nagasaki University, Sakamoto, Nagasaki, Japan; 2 Faculty of Pharmacy, University of Aleppo, Aleppo, Syria; 3 Institute of Research and Development, Duy Tan University, Da Nang, Vietnam; 4 School of Medicine and Pharmacy, Duy Tan University, Da Nang, Vietnam

As of 13 October 2022, Malaysia’s reported coronavirus disease 2019 (COVID-19) cases surpassed 4.8 million [1]. Up to that date, almost 65 million tests have been conducted, and the disease has claimed the lives of 36 410 people since its first detection in the country. Furthermore, the disease caused 7082 clusters, of which only 13 remained active as of October 2022. The disease trend in Malaysia in 2022 shows that beginning from February, there was a spike in the number of daily reported cases driven by the Omicron variant, which peaked on 5 March with 33 406 new cases. Almost a week later, the trend saw a sharp decrease and continued decreasing to reach as low as 922 new cases on 3 May. As the government announced that the country would shift to the endemic phase and started easing restrictions accordingly, the cases steadily increased again after the 3 May but not surpassing 6000 new cases on any day as of 13 October.

Throughout the pandemic, the Malaysian government imposed various strict state and border control mandates. The enforcement of movement control brought down cases to significantly low numbers [2]. Only some businesses were allowed to operate within limited working hours. Working from home was practised in the government and private sector. Non-essential businesses and social activities were not allowed. Public gathering was prohibited, and schools were closed intermittently. Wearing masks became mandatory on 1 August 2020, and those who did not adhere were subject to a fine [3]. Rapidly detecting and terminating clusters was one of the government’s main priorities. They developed a smartphone application called “MySejahtera” to conduct contact tracing and rapidly identify suspected cases who came into contact with positive cases. Using this application was mandatory, and only healthy people were allowed to check in into premises. Targeted, instead of mass, testing was conducted in high-risk areas [4]. As for the clinical management of confirmed cases, a guideline issued by the Ministry of Health (MOH) and updated on 31 May 2022, recommends using, based on the patient’s clinical stage, antiviral, corticosteroid or anticoagulation therapy [5].

The National COVID-19 immunisation program is the Malaysian government’s initiative to provide free vaccination to all residents of Malaysia regardless of their nationality. The program took off on 24 February 2021 and sequentially targeted frontline workers, high-risk individuals, and adults over 18 years old [6]. As of 13 October 2022, 86.1% and 84.2% of the population had received the first and second doses of a COVID-19 vaccine. Additionally, 49.7% received one booster shot, and 1.6% received a second one. The Pfizer–BioNTech vaccine accounted for the largest share (61.2%), followed by Sinovac’s (29.8%), AstraZeneca’s (7.9%), and CanSino’s (0.3%). The government created new enforcements to encourage more people to get vaccinated, such as allowing only vaccinated people to enter shops, malls, and restaurants.

Over two years into the COVID-19 pandemic, Malaysia’s aggressive response to the outbreak was crowned with success. In March 2022, the prime minister announced that the country would shift into the endemic phase in April [7]. Even though the number of cases was rising in the country due to the Omicron variant wave, the government felt it was time to restore full operation hours for businesses, ease social distancing measures and reopen international borders. This was driven by the fact that only 0.7% of the cases were severe, and 98.7% of the adults became fully vaccinated. As of 1 May 2022, wearing facemasks outdoors became optional, while it remained mandatory in closed places and public transportation [8]. The transition into the endemic phase was manifested by the transition in the government’s narrative from enforcing stringent containment measures to depending on community solidarity and individual responsibility, as put by its health minister.

The Malaysian government has remained at the vanguard of responding to this menace since its emergence. Lockdowns, movement restrictions, enforcement of public health measures, limited public gatherings, rapid contact tracing, targeted testing, free severe and widespread coverage, utilisation of technology, support of non-governmental organisations and the high adherence of the public contributed to Malaysia’s success. Although it was seen as draconian at some point, it was paramount to maintain a low infection rate. Containment measures must be revised regularly, following the current trends and considering the burden on a country’s health care system. COVID-19 will remain present for a long time; hence, governments must learn to adapt, stay vigilant and act swiftly when needed, as prolonged border closures, movement control, lockdowns, and restricted operating models and hours will only have a deleterious impact on the economy and the livelihood of the citizens. Nations worldwide can draw lessons from the successful experience of Malaysia in controlling the now endemic COVID-19.
